# Acute aerobic exercise alters serum protein distribution in colorectal cancer patients

**DOI:** 10.3389/fonc.2025.1586344

**Published:** 2025-04-09

**Authors:** Sinan Jin, Shude Han, Ning Wang, Mingrui Yang, Chao Chen

**Affiliations:** ^1^ Department of Pathology, First Affiliated Hospital of Harbin Medical University, Harbin, China; ^2^ Department of Comprehensive Rehabilitation, Beidahuang Industry Group General Hospital, Harbin, China; ^3^ Department of Gastroenterological Surgery, Beidahuang Industry Group General Hospital, Harbin, China

**Keywords:** colorectal cancer, acute aerobic exercise, proteomics, differential expression analysis, WGCNA

## Abstract

**Background:**

Acute aerobic exercise has been shown to exert beneficial effects on colorectal cancer (CRC) patients, however, the specific molecular mechanisms underlying these effects remain unclear. To investigate the relationship between exercise and CRC progression, we conducted a prospective cohort study to analyze the impact of acute aerobic exercise on serum protein profiles in CRC patients.

**Methods:**

Serum samples from 10 CRC patients were collected and analyzed using proteomics following either no exercise or acute aerobic exercise. Weighted gene co-expression network analysis (WGCNA) was employed to identify protein modules associated with exercise. Protein-protein interaction (PPI) analysis was further conducted to pinpoint key proteins influenced by exercise. Western blotting was used to validate the expression changes of identified proteins.

**Results:**

WGCNA revealed that the blue module exhibited the highest correlation with 42 serum protein, 27 of which showed significant changes post-exercise compared with pre-exercise. PPI analysis identified ARF6, ARF5, and RAB11A as the core proteins. Western blotting further confirmed that their expression levels were significantly reduced in the post-exercise group, making them key targets in current clinical treatment protocols.

**Conclusion:**

This study demonstrates that acute aerobic exercise alters the serum protein profile in CRC patients, with significant reductions in ARF6, ARF5, and RAB11A representing the most meaningful changes. These findings provide strong evidence supporting the use of acute aerobic exercise as a therapeutic intervention for CRC.

## 1 Introduction

Colorectal cancer (CRC) ranks as the third most predominant cancer worldwide and is the leading cause of cancer-related fatalities. It is acknowledged to be a prevalent international health crisis (1). The prevalence is anticipated to increase by 60% by 2030 (2). Immunotherapy, gene therapy, and emerging lymph node clearance techniques have shown remarkable anti-tumor effects (3–6). Although patient survival has improved, pain treatment and fear of the disease disrupt patients’ lifestyles (7, 8). In fact, the high prevalence of CRC is closely related to chronic inflammatory bowel disease and lifestyle, including factors such as physical inactivity, obesity, and an unhealthy diet (9, 10).

Physical activity has been demonstrated to significantly mitigate the risk and progression of chronic diseases, including colorectal cancer (CRC) (11). Evidence suggests that higher levels of physical activity are associated with a marked reduction in mortality among CRC survivors (10). Furthermore, exercise has been shown to enhance quality of life, reduce CRC-specific mortality, and lower all-cause mortality in both CRC patients and survivors (12, 13). Defined as a structured and purposeful form of movement aimed at improving physical fitness (14), exercise induces the release of biologically active molecules, such as proteins, nucleic acids, and metabolites, from skeletal muscles and other glands into the bloodstream. These molecules can exert systemic effects on distant cells through endocrine-like signaling mechanisms (15). While different types of physical activity may be beneficial for various cancer types (16), research in this area remains limited. Notably, few randomized controlled trials have explored the impact of high-intensity aerobic exercise in CRC patients, and existing studies are often constrained by small sample sizes (17).

We developed an acute aerobic exercise at a certain intensity as a training program for patients with CRC. The serum proteome was analyzed by extracting the serum from patients before and after acute aerobic exercise. We obtained overlapping proteins of the two analyses using two popular analytical methods, differential protein analysis and weighted gene co-expression network analysis (WGCNA), to observe specific differential proteins before and after exercise. Proteins play crucial roles in various biological processes, molecular functions, and creation of cellular components. Their participation is essential and widespread, making them significant contributors to biology. Progress in cancer immunotherapy has emphasized the critical requirement for biomarkers to forecast reactions to immune checkpoint obstruction and to select new antigens for individualized vaccine production (18). Proteomics offers novel solutions for these requirements. Our study presents data on biomarkers related to acute aerobic exercise at specific intensities, and potentially provides compelling evidence for the beneficial impact of exercise on CRC patients.

## 2 Methods

### 2.1 Participants

We recruited patients diagnosed with CRC between January 2023 and June 2023. During the history-taking process, a questionnaire was used to determine the patients’ exercise. The inclusion criteria were age ≥50 years, body mass index (BMI) ≥18.4, and never engaged in moderate-to-vigorous physical activity standardized as ≥3 days in 3 months and ≥30 minutes per week (19). No treatment for any type of malignant tumor. Not receiving insulin or an insulin sensitizer and having no risk factors (such as cardiopulmonary disease, previous myocardial infarction, and low ejection fraction) that would prevent the safe completion of the exercise study. Participants were required to obtain a doctor’s consent to participate in the program. This study was approved by the Ethics Committee of Beidahuang Industry Group General Hospital (No. KY-2023101801), and informed consent was obtained from all participants. None of the participants had died before the end of the experiment.

### 2.2 Experimental models of exercise

Ten CRC patients performed moderate-intensity interval aerobic exercise on a cardiopulmonary exercise testing machine (COSMED, Quark PFT Ergo, Italy) (19). The entire procedure was performed under the supervision of researchers from the Department of Comprehensive Rehabilitation of Beidahuang Industry Group General Hospital. After a 10-minute warm-up of low-resistance pedaling (30 W), patients performed 6×5 minutes of interval exercise at 65%-75% of their maximal heart rate. They were assessed for perceived exertion every 5 min of exercise, with 3 min of active recovery between each set (20). The patients exercised for 30 min. Blood samples were collected immediately after completion of the exercise program.

### 2.3 No experimental models of exercise

To control for natural bias in serum analysis, control serum samples were collected from the same 10 CRC patients, as described above. On the day of testing, control serum was collected early in the morning. Patients were placed on an empty stomach and did not exercise. This eliminates the possibility that other variables may have influenced the results.

### 2.4 Serum preparation

Blood samples, each approximately 20 ml, were collected from the antecubital vein using four 5 ml Vacutainer serum tubes (BD, South Carolina). After collection, the samples were left to clot at room temperature for approximately 30 minutes. They were then centrifuged at 1000 × g for 15 minutes to separate the serum, which was carefully aliquoted and stored at -80°C until further analysis.

### 2.5 Sample preparation for proteome analysis

Proteomic analyses were performed uniformly after collecting 20 serum samples from the 10 patients. Technical support for proteomics was provided by the Shanghai Applied Protein Technology Co. In total, low-abundance proteins in each serum/plasma sample were obtained using magnetic beads (21). An in-solution digestion procedure was used to digest the proteins bound to magnetic beads. The peptide content was estimated using the UV light spectral density at 280 nm. For data independent acquisition (DIA) experiments, iRT calibration peptides were spiked into each sample. Equal amounts of digested peptides were pooled into one sample and fractionated using the Thermo Scientific™ Pierce™ High pH Reversed-Phase Peptide Fractionation Kit. Each fraction was desalted and re-constituted.

### 2.6 Mass spectrometry assay for DIA and MS data analysis

The peptides from each sample were analyzed using a TIMSTOF mass spectrometer (Bruker) connected to an Evosep One system liquid chromatograph (Denmark) in DIA mode. The mass spectrometer collected the ion mobility MS spectra over a mass range of m/z 100-1700. The DIA data were analyzed using Spectronaut™ 16. This database was downloaded from http://www.uniprot.org/. The iRT peptide sequence was added (BiognosysiRT Kit). All results were filtered based on a value cutoff of 0.01 (equivalent to FDR<0.01).

### 2.7 Bioinformatic analysis

Cluster 3.0 (http://bonsai.hgc.jp/~mdehoon/software/cluster/software.htm) and Java Treeview software (http://jtreeview.sourceforge.net) were used to perform the hierarchical clustering analysis.

#### 2.7.1 GO and KEGG annotation

The protein sequences of the selected differentially expressed proteins were locally searched to find homologous sequences, gene ontology (GO) terms were mapped, and sequences were annotated using the software program Blast2GO. Proteins were blasted against the online Kyoto Encyclopedia of Genes and Genomes (KEGG) database (https://www.genome.jp/kegg/) to retrieve their KEGG orthology identifications, and were subsequently mapped to KEGG pathways (22–24).

#### 2.7.2 Enrichment analysis

Enrichment analyses were applied based on Fisher’s exact test, considering all quantified proteins as the background dataset. The Benjamini- Hochberg correction for multiple testing was further applied to adjust the derived p-values. Only functional categories and pathways with p-values under a threshold of 0.05 were considered significant (25–27).

#### 2.7.3 Protein-protein interaction analysis

PPI information of the studied proteins was retrieved from the IntAct molecular interaction database (http://www.ebi.ac.uk/intact/) using their gene symbols or STRING software (http://string-db.org/). The results were downloaded and imported into the Cytoscape software (http://www.cytoscape.org/, version 3.2.1). The degree of each protein was calculated to evaluate the importance of the proteins in the PPI network.

#### 2.7.4 Weighted gene co-expression network analysis

Weighted Gene Co-Expression Network Analysis (WGCNA) was performed using the WGCNA package in R (Version 1.69) to identify distinct protein modules among all detected proteins. A weighted protein co-expression network was constructed based on the log2-transformed protein abundance matrix (28).

### 2.8 Western Blot analysis

The Western Blot assay was conducted following established protocols (29–31). High-abundance proteins were first removed, and five pairs of serum samples were randomly selected for analysis. Serum protein samples were diluted and quantified using the BCA method. Subsequently, the samples were subjected to electrophoresis on SDS-PAGE gels, followed by transfer to PVDF membranes. The membranes were blocked with 5% fetal bovine serum and incubated with the following primary antibodies: ARF6 (20225-1-AP, Proteintech, China), ARF5 (20227-1-AP, Proteintech, China), RAB11A (20229-1-AP, Proteintech, China), and transferrin (66171-1-lg, Proteintech, China) (32). After incubation with species-specific secondary antibodies for 1 hour at room temperature, protein expression levels were visualized using the ECL detection method.

### 2.9 Statistical analysis

Western Blot experiments were repeated at least three times, and the results were calculated as gray values using ImageJ (33). Statistical analyses were performed using Prism 8.0.2. Comparisons between the two groups were made using the t-test, and statistical results were expressed as mean ± SEM values, with *P* < 0.05 considered statistically significant.

## 3 Results

### 3.1 Acute aerobic exercise alters serum protein expression levels in CRC patients

**Figure 1:**
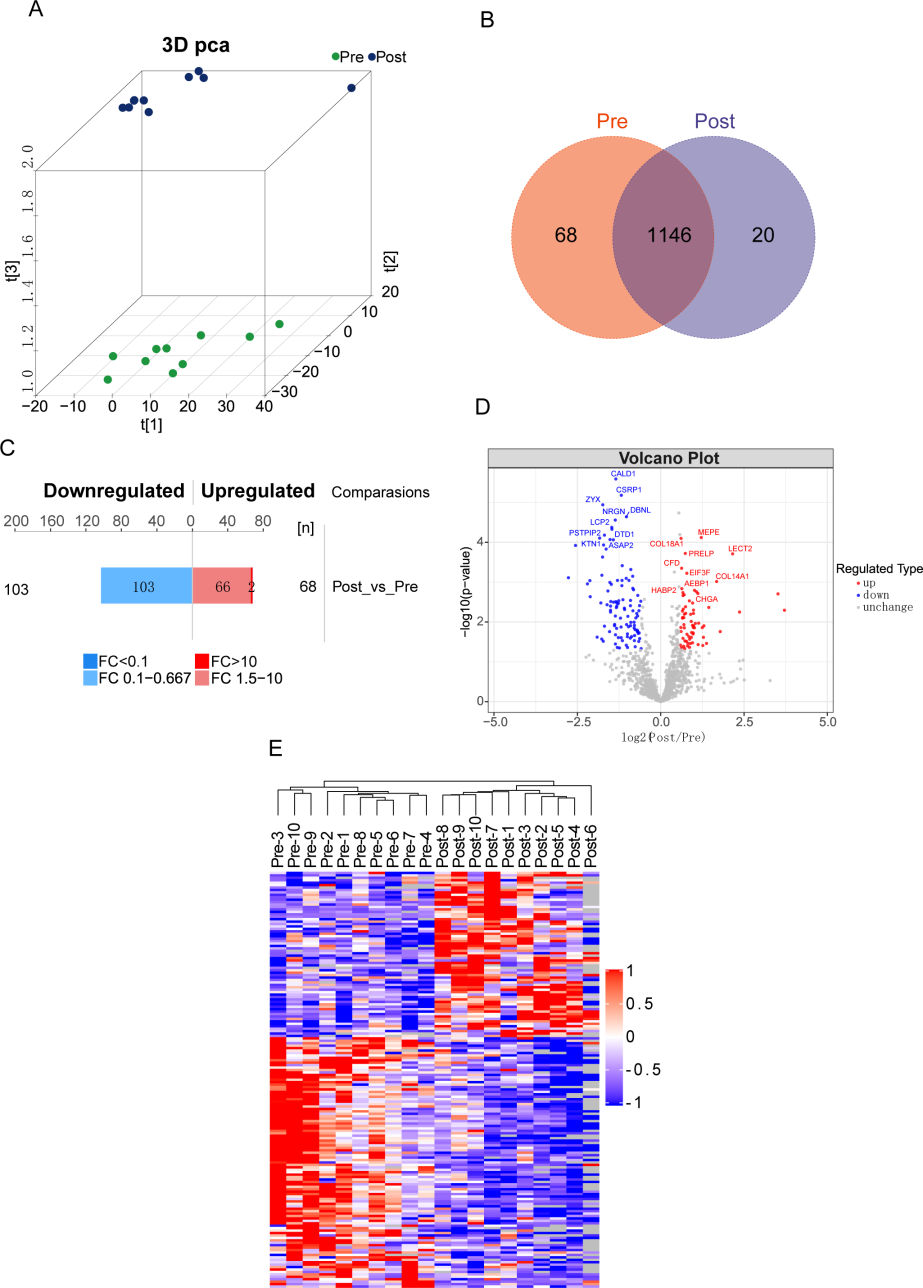
Differential expression in serum proteomes before and after acute aerobic exercise in CRC patients. **(A)** Aggregation of principal components in serum of CRC patients before and after experiencing acute aerobic exercise. **(B)** Venn diagram showing 1146 overlapping proteins in the two groups. **(C)** Histograms and **(D)** volcano plots showing the number and names of significantly differentially expressed proteins between Pre and Post groups. **(E)** Heatmap of hierarchical clustering analysis of protein expression in each sample. (Pre: before experiencing acute aerobic exercise, Post: after experiencing acute aerobic exercise).

The baseline characteristics of the 10 colorectal cancer (CRC) patients who participated in the acute aerobic exercise trial are summarized in **Table 1**. A three-dimensional (3D) principal component analysis (PCA) demonstrated distinct separation between the post-exercise (Post) and pre-exercise (Pre) groups, indicating significant differences in their principal components (**Figure 1A**). Additionally, a total of 1,146 overlapping proteins were identified across the samples (**Figure 1B**). To screen for significantly differentially expressed proteins (DEPs), a fold change (FC) threshold of >1.5 was defined as upregulation and <0.67 as downregulation, with a p-value <0.05 considered statistically significant (34). Among the 171 DEPs identified, 103 were downregulated and 68 were upregulated following exercise (**Figure 1C**). Volcano plots further illustrated the differential expression patterns of selected DEPs, highlighting their respective fold changes (**Figure 1D**; Supplementary Table 1). Hierarchical clustering analysis provided a more intuitive picture of the serum protein expression in each patient. As shown in **Figure 1E**, acute aerobic exercise induced notable alterations in the distribution of serum proteins.

**Table 1:** Patients in this study - basic information.

Characteristic	Mean ± SD or number (%)
Gender Male Female	4 (40) 6 (60)
Age (y)	63.00 ± 8.08
Body mass (kg)	63.58 ± 10.94
Height (cm)	162.60 ± 8.72
BMI (kg/m^2^)	24.02 ± 2.89
Smoking status Current smoker Previous smoker	0 (0) 3 (30)
Ethnicity Han Chinese people	10 (100)
Marital status Married Single	7 (70) 3(30)
Chemotherapy No chemotherapy	10(100)

### 3.2 Functional properties of differentially expressed proteins

**Figure 2:**
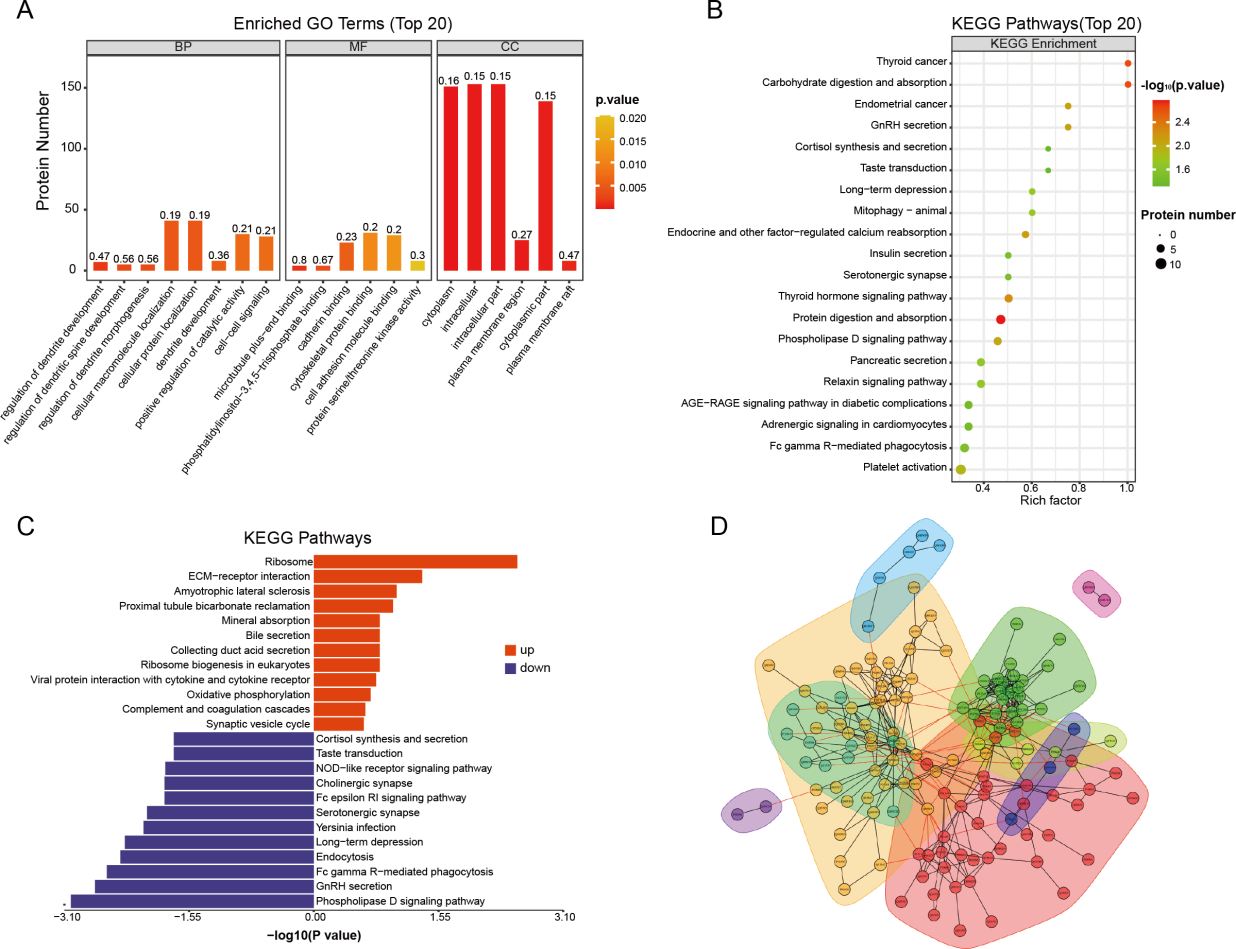
Functional enrichment analysis of differential proteins before and after acute aerobic exercise. **(A)** GO enrichment analysis of 171 differentially expressed proteins (BP, biological processes; MF, molecular functions and CC, cellular components). The vertical coordinates represent the number of different proteins under each functional classification. The color bars represent the importance of the enriched GO functional classifications. **(B)** KEGG pathway analysis of 171 differentially expressed proteins. **(C)** Butterfly plot showing the enrichment of up-regulated (red or right) and down-regulated (blue or left) KEGG pathways. **(D)** Plot of interaction patterns of significantly different proteins between groups of Post *vs*. Pre. Annotated with different colored backgrounds based on similar biological functions.

We initially conducted Gene Ontology (GO) functional analysis (p-value <0.05) to enrich and categorize the 171 differentially expressed proteins (DEPs). **Figure 2A** illustrates the involvement of DEPs in biological processes (BP), molecular functions (MF), and cellular components (CC). The analysis revealed that acute aerobic exercise predominantly alters the expression of cytoplasmic proteins, which are primarily associated with the localization of cellular macromolecules and the positive regulation of catalytic activity. In terms of MF, the DEPs were found to predominantly bind to cadherin and cytoskeletal proteins. Subsequently, Kyoto Encyclopedia of Genes and Genomes (KEGG) pathway analysis (P <0.05) was employed to identify enriched pathways among the 171 DEPs (**Figure 2B**). The results indicated that these DEPs are mainly involved in protein and carbohydrate digestion and absorption, as well as thyroid cancer. To further explore the functional relevance of these pathways, KEGG pathway enrichment analysis was performed separately for the up- and down-regulated DEPs (**Figure 2C**). The butterfly plots demonstrated that up-regulated DEPs were primarily associated with ribosome and extracellular matrix (ECM)-receptor interactions, whereas down-regulated DEPs were predominantly linked to the phospholipase D signaling pathway. Finally, protein-protein interaction (PPI) analysis was conducted, revealing direct interactions among 140 out of the 171 proteins. Based on their high aggregation and functional similarity, these proteins were classified into five distinct groups (**Figure 2D**).

### 3.3 CRC patients’ serum protein can be divided into 9 modules

**Figure 3:**
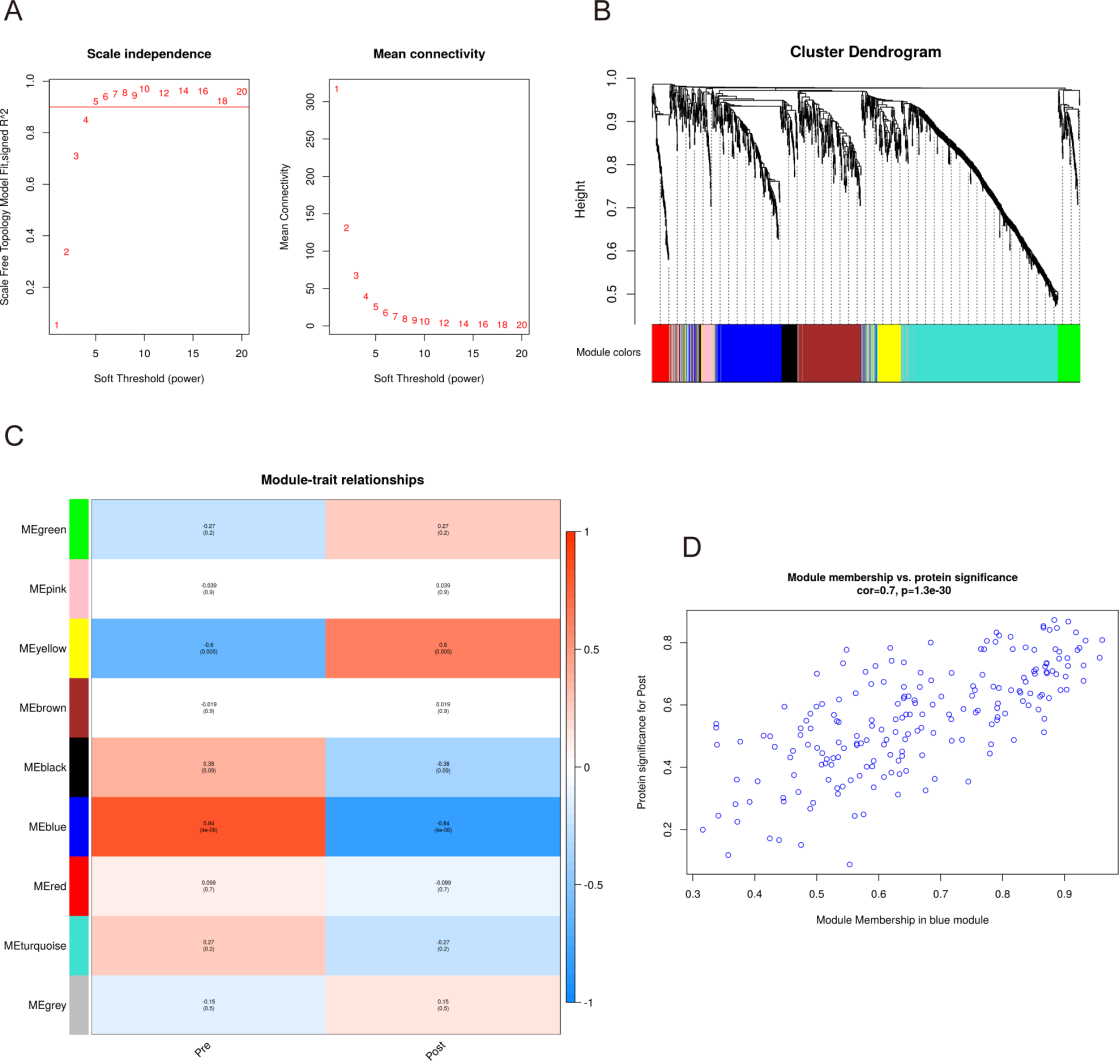
WGCNA of the serum proteome before and after acute aerobic exercise. **(A)**The horizontal coordinates of both graphs, represent the value of the soft threshold (power) taken. The vertical coordinate of the left graph is the scale-free fit index, i.e., signed R2, and the vertical coordinate of the right graph represents the average connectivity of all nodes. **(B)** The dendrogram shows the clustering of proteins and the colours below represent the nine identified modules. **(C)** Module trait relationships for clinical traits. The left side of each cell displays the module name and shows the correlation between the module trait genes and each trait. A colour-coded table indicates the strength of the correlation. **(D)** Scatterplot showing the relationship between protein importance and module membership in the blue module.

To detect the co-expressed proteins, a weighted co-expression network based on 1181 filtered proteins was constructed using WGCNA. Two clinical parameters, namely pre-exercise and post-exercise, were utilized in the application of WGCNA. To build the scale-free network, we established a soft-threshold power β of 5, set the independence at 0.9, and maintained the average connectivity near 0 (**Figure 3A**). Calculation of expression correlation coefficients between proteins based on the optimal soft threshold identified nine co-expression modules that were generated: green, pink, yellow, brown, black, blue, red, turquoise, and gray (**Figure 3B**). The gray module contains proteins that cannot be merged into any of the other modules. It also shows the co-expression of pre- and Post as traits.

### 3.4 Functional properties of proteins in blue modules

As shown in **Figure 3C**, the blue module had the highest correlation with the presence or absence of acute aerobic exercise, and the proteins within the module were negatively correlated post-exercise (cor=-0.84, *p*-value=4×10^-6^). In addition, consistency analysis showed a high correlation between the blue module and the proteins detected in the patient serum (**Figure 3D**). Therefore, we selected the blue module from the nine available modules for further analysis.

**Figure 4:**
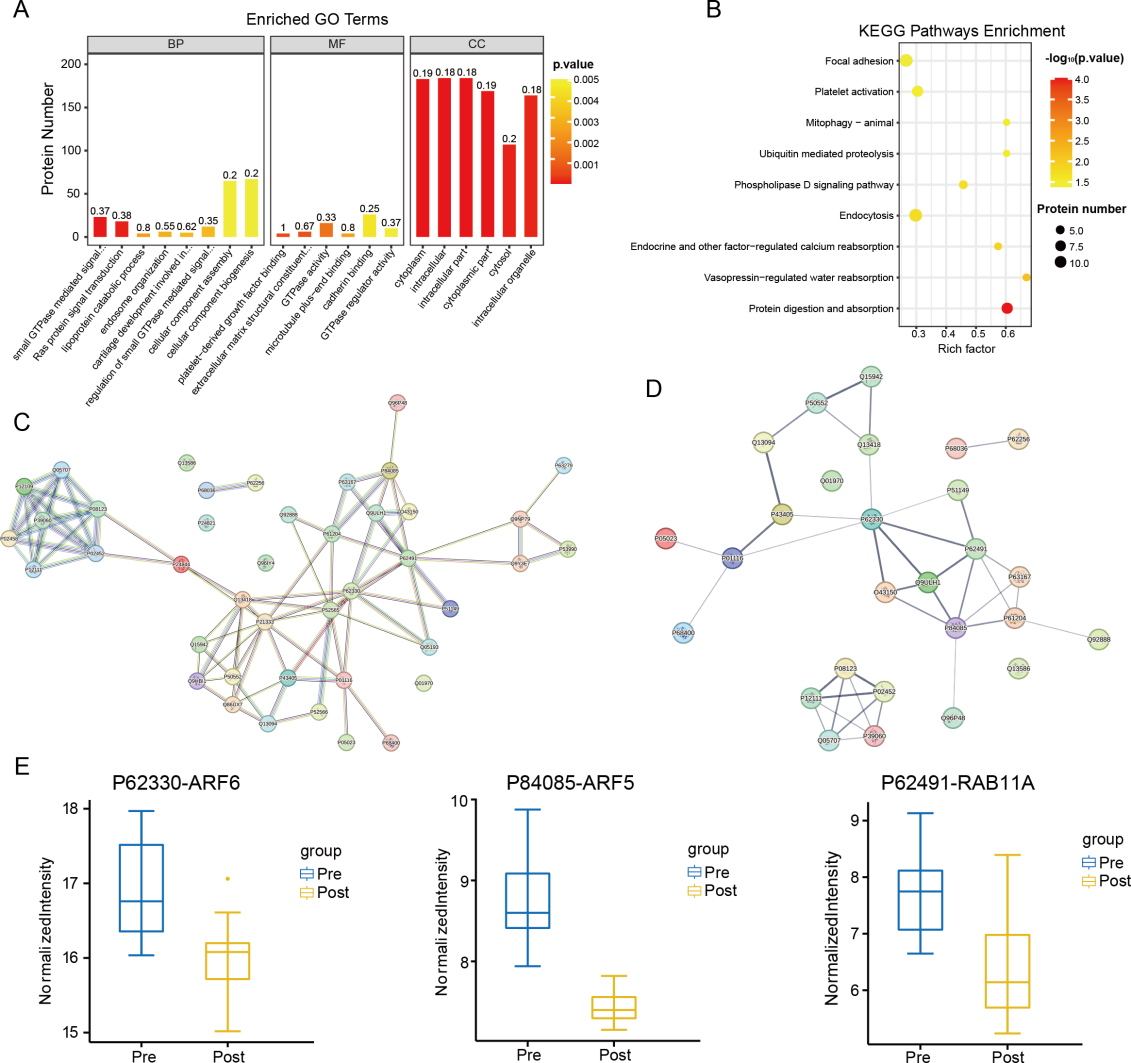
Enrichment analysis. **(A)** GO enrichment analysis at P value < 0.05 under the blue module of WGCNA. **(B)** KEGG pathway enrichment analysis under the blue module of WGCNA at P value < 0.05. **(C)** PPI analysis result of the 42 proteins (When the p-value of GO terms and KEGG pathways was less than 0.05, there were the same 42 proteins). **(D)** Results of PPI analysis for 27 proteins that were significantly differentially expressed among the 42 proteins. **(E)** Extracting the results of proteomic analysis of CRC patients, it can be seen that the levels of ARF6, ARF5 and RAB11A were decreased in the Post group (ARF6, ARF5 and RAB11A have the highest connectivity in the PPI analysis).

Gene Ontology (GO) analysis was conducted on the 199 proteins included in the blue module (**Figure 4A**). These proteins are predominantly localized intracellularly and within the cytoplasm, with their primary molecular function being the regulation of GTPase activity. Additionally, they play a role in modulating Ras signaling pathways. Kyoto Encyclopedia of Genes and Genomes (KEGG) pathway enrichment analysis was also performed on these 199 proteins (**Figure 4B**). Consistent with the earlier KEGG analysis of differentially expressed proteins (DEPs), the proteins in the blue module were primarily associated with the phospholipase D signaling pathway and protein digestion and absorption processes. After strictly controlling the *P*-values of the GO and KEGG enrichment pathways to be less than 0.05, 42 proteins appeared simultaneously in the two enrichment methods. Next, the proteins were subjected to protein-protein interaction (PPI) analysis (**Figure 4C**).

### 3.5 Acute aerobic exercise significantly reduces serum levels of ARF6, ARF5 and RAB11A in patients with CRC

By intersecting the differentially expressed proteins (DEPs) identified in the Post *vs*. Pre comparison with the protein-protein interaction (PPI) proteome from the blue module of Weighted Gene Co-Expression Network Analysis (WGCNA), we identified 27 proteins. PPI analysis was subsequently performed on these proteins (**Figure 4D**). The results revealed that three proteins exhibited the highest number of interactions: P62330 (ARF6) interacted with seven proteins, while P84085 (ARF5) and P62491 (RAB11A) each interacted with six proteins. These three proteins were also observed among the DEPs, with all showing a significant decrease in expression (**Figure 4E**).

**Figure 5:**
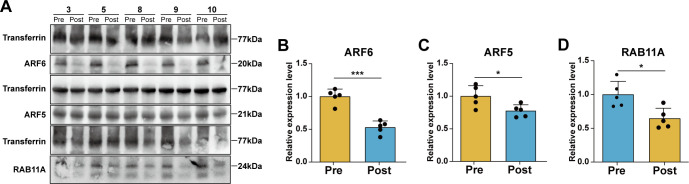
Changes of protein expression levels in serum of CRC patients before and after aerobic exercise. **(A)** Representative images of the expression levels of ARF6, ARF5, RAB11A and the internal reference protein Transferrin. Statistical analysis of relative expression levels of **(B)** ARF6, **(C)** ARF5 and **(D)** RAB11A (n=5, compared with the Pre group ^*^ *P* < 0.05, ^***^ *P* < 0.001).

To further investigate the changes in expression levels of ARF6, ARF5, and RAB11A before and after exercise in patients, serum protein samples from five randomly selected patients were subjected to Western blotting (**Figure 5A**). The results demonstrated a reduction in the expression levels of all three proteins in the Post group compared to the Pre group (**Figures 5B-D**). Collectively, both the proteomic analysis and Western blotting experiments indicated that the serum levels of ARF6, ARF5, and RAB11A in CRC patients significantly decreased following acute aerobic exercise.

## 4 Discussion

Multi-omics has been widely used in tumor diagnosis and detecting potential biomarkers (35–38), such as single-cell analysis and machine learning (39–43). In this study, we employed advanced proteomic and bioinformatics techniques to investigate changes in serum protein levels in colorectal cancer (CRC) patients before and after acute aerobic exercise. Using differential expression analysis, we identified 171 significantly altered proteins, while weighted gene co-expression network analysis (WGCNA) revealed 42 proteins strongly associated with the blue module. These findings suggest that acute aerobic exercise may influence protein distribution in CRC patients, potentially modulating disease-related pathways. The identified differentially expressed proteins (DEPs) provide valuable insights into the mechanisms underlying the effects of exercise and could serve as a foundation for further research into its therapeutic potential in CRC.

By intersecting the 171 differentially expressed proteins (DEPs) identified through differential expression analysis with the 42 core proteins from weighted gene co-expression network analysis (WGCNA), we obtained 27 specific proteins. These proteins not only validate the reliability of both methods but also highlight their potential functional significance in acute aerobic exercise among CRC patients. WGCNA identifies proteins with similar expression patterns, revealing functional modules and interactions (44), while differential expression analysis focuses on proteins significantly altered under specific conditions, pinpointing key players in acute aerobic exercise (45). Combining these approaches enhances the identification of regulatory networks and molecular mechanisms underlying biological processes. Among the 27 proteins analyzed in the protein-protein interaction (PPI) network, ARF6, ARF5, and RAB11A emerged as central nodes due to their high connectivity. Differential expression analysis revealed a significant decrease in their levels post-exercise, which was further confirmed by Western blotting. These findings suggest that ARF6, ARF5, and RAB11A may play critical roles in mediating the effects of acute aerobic exercise in CRC.

There are three classes of Arf GTPases: class I (ARF1 and -3), class II (ARF4 and -5), and class III (ARF6) (46). Previous research has indicated that ARF6 has a significant impact on tumor proliferation, angiogenesis, invasion, metastasis, and immune evasion (47, 48). A study on the inhibition of metastasis in CRC cells revealed that the invasive ability of tumor cells decreased upon ARF6 inhibition; conversely, it increased when ARF6 was overexpressed (49). One study found that elevated ARF6 promotes hepatocellular carcinoma (HCC) metastasis (50). Tumor-related analysis of ARF5 has been limited thus far. A small number of studies have addressed ARF5 interaction with Rab11-FIP4, and ARF5 overexpression greatly increased the number and size of HCC spheres. Simultaneously, depletion of ARF5 dramatically decreases the stemness of HCC cells and improves their sensitivity to cancer therapeutic agents (51). These studies suggest that the expression levels of ARF6 and ARF5 are positively correlated with the invasive ability of cancer cells. In this study, the expression levels of ARF6 and ARF5 in the serum of CRC patients were significantly reduced after exercise, reflecting the positive intervention effect of acute aerobic exercise on cancer patients.

The Rab11 family, consisting of the RAB11A, RAB11B, and RAB25 proteins, is a key regulator of exocytic and recycling processes. They can regulate protein and vesicle formation and transport from early and recycled endosomes to the cell surface (52). Despite sharing high sequence homology, the RAB11B protein is mainly expressed in the brain, heart, and testis, RAB25 expression is restricted to epithelial cells, and RAB11A is expressed ubiquitously (53, 54). In a study on breast cancer, RAB11A overexpression accelerated tumor cell viability, migration, and invasiveness. In a prostate cancer study, RAB11A inhibition was found to attenuate cancer cell proliferation and invasiveness. Combined with previous studies, we expect that RAB11A has great potential for further studies on CRC.

ARF6, ARF5, and RAB11A belong to different small GTPase superfamily (55, 56). However, they are considered therapeutic targets that cannot be treated with drugs because of their complex regulatory mechanisms and lack of ligand-binding space (57, 58). Our research provides new insights into this area and suggests that acute aerobic exercise may be a way to regulate the expression of these proteins.

With significant progress in the clinical treatment of cancer, the survival time of patients has increased; however, new problems have arisen. How should they face life? Should they sit still or exercise moderately? Scientists have also realized this problem, and research on the lifestyles of cancer patients has become an important topic. Research suggests that muscle contraction is an immune modulator that may enhance the immune response against cancer (59). The cytokines produced by muscles during exercise are important factors in maintaining a healthy immune effector cell population and promoting an overall anti-inflammatory environment (31, 33, 60, 61). The basis of many cancers is a systemic low-grade chronic inflammation (62). Therefore, youth and exercise may be key factors in fighting some cancers.

Research indicates that acute exercise lasting ≥20-60 minutes mobilizes lymphocytes (63). Initially, increased blood pressure and shear stress trigger the release of lymphocytes from blood vessels and tissue reservoirs (e.g., lungs, liver, and spleen), elevating white blood cell counts in peripheral circulation (64). Subsequently, adrenergic and noradrenergic pathways are activated, modulating receptors on lymphocyte surfaces (59). Our study supports the inclusion of physical activity as a complementary approach to clinical treatment for cancer patients. However, further research is necessary to determine the optimal exercise regimens for specific diseases. Additionally, individualized assessment of patient status during clinical treatment is essential to develop tailored exercise strategies.

Our study had some limitations. First, the sample size was small and no more patients were eligible to participate in the experiment. Currently, there are 20 serum samples from 10 patients, and more patient samples may be needed to increase power. On the other hand, the specific differential proteins ultimately obtained by taking the intersection of the two analyses need to study all the specific differential proteins in their entirety. We validated and preliminarily explored three highly associated proteins, and further studies are needed to verify their functional and mechanistic importance. In future studies, we will collect more patient groups, as well as the full range of validation and exploration of specific differentially expressed proteins, which may shed more light on the intervention angle and effect of acute aerobic exercise on CRC and provide more comprehensive and powerful evidence for the effect of acute aerobic exercise on CRC.

## Data Availability

The original contributions presented in the study are included in the article/supplementary material. Further inquiries can be directed to the corresponding authors.

## References

[1] Sung H Ferlay J Siegel RL Laversanne M Soerjomataram I Jemal A. Global cancer statistics 2020: GLOBOCAN estimates of incidence and mortality worldwide for 36 cancers in 185 countries. CA Cancer J Clin. (2021) 71:209–49. doi: 10.3322/caac.21660 33538338

[2] Eng C Yoshino T Ruiz-Garcia E Mostafa N Cann CG O’Brian B. Colorectal cancer. Lancet. (2024) 404:294–310. doi: 10.1016/S0140-6736(24)00360-X 38909621

[3] Xia Z Chen S He M Li B Deng Y Yi L. Editorial: Targeting metabolism to activate T cells and enhance the efficacy of checkpoint blockade immunotherapy in solid tumors. Front Immunol. (2023) 14:1247178. doi: 10.3389/fimmu.2023.1247178 37575246 PMC10415066

[4] Zhang X Zhang P Cong A Feng Y Chi H Xia Z. Unraveling molecular networks in thymic epithelial tumors: deciphering the unique signatures. Front Immunol. (2023) 14:1264325. doi: 10.3389/fimmu.2023.1264325 37849766 PMC10577431

[5] Deng Y Shi M Yi L Naveed Khan M Xia Z Li X. Eliminating a barrier: Aiming at VISTA, reversing MDSC-mediated T cell suppression in the tumor microenvironment. Heliyon. (2024) 10:e37060. doi: 10.1016/j.heliyon.2024.e37060 39286218 PMC11402941

[6] Zhou JG Liang R Wang HT Jin SH Hu W Frey B. Identification and characterization of circular RNAs as novel putative biomarkers to predict anti-PD-1 monotherapy response in metastatic melanoma patients - Knowledge from two independent international studies. Neoplasia. (2023) 37:100877. doi: 10.1016/j.neo.2023.100877 36696838 PMC9879779

[7] Lim CYS Laidsaar-Powell RC Young JM Kao SC Zhang Y Butow P. Colorectal cancer survivorship: A systematic review and thematic synthesis of qualitative research. Eur J Cancer Care (Engl). (2021) 30:e13421. doi: 10.1111/ecc.13421 33733545

[8] El-Shami K Oeffinger KC Erb NL Willis A Bretsch JK Pratt-Chapman ML. American cancer society colorectal cancer survivorship care guidelines. CA Cancer J Clin. (2015) 65:428–55. doi: 10.3322/caac.21286 PMC5385892 26348643

[9] Siegel RL Wagle NS Cercek A Smith RA Jemal A. Colorectal cancer statistics, 2023. CA Cancer J Clin. (2023) 73:233–54. doi: 10.3322/caac.21772 36856579

[10] Roshandel G Ghasemi-Kebria F Malekzadeh R. Colorectal cancer: epidemiology, risk factors, and prevention. Cancers (Basel). (2024) 16(8):1530. doi: 10.3390/cancers16081530 38672612 PMC11049480

[11] Cormie P Zopf EM Zhang X Schmitz KH. The impact of exercise on cancer mortality, recurrence, and treatment-related adverse effects. Epidemiol Rev. (2017) 39:71–92. doi: 10.1093/epirev/mxx007 28453622

[12] Balhareth A Aldossary MY McNamara D. Impact of physical activity and diet on colorectal cancer survivors’ quality of life: a systematic review. World J Surg Oncol. (2019) 17:153. doi: 10.1186/s12957-019-1697-2 31472677 PMC6717629

[13] Austin PD Lee W Costa DS Ritchie A Lovell MR. Efficacy of aerobic and resistance exercises on cancer pain: A meta-analysis of randomised controlled trials. Heliyon. (2024) 10:e29193. doi: 10.1016/j.heliyon.2024.e29193 38623224 PMC11016720

[14] Dasso NA. How is exercise different from physical activity? A concept analysis. Nurs Forum. (2019) 54:45–52. doi: 10.1111/nuf.2019.54.issue-1 30332516

[15] Chow LS Gerszten RE Taylor JM Pedersen BK van Praag H Trappe S. Exerkines in health, resilience and disease. Nat Rev Endocrinol. (2022) 18:273–89. doi: 10.1038/s41574-022-00641-2 PMC9554896 35304603

[16] Jee H Park E Hur K Kang M Kim Y. High-intensity aerobic exercise suppresses cancer growth by regulating skeletal muscle-derived oncogenes and tumor suppressors. Front Mol Biosci. (2022) 9:818470. doi: 10.3389/fmolb.2022.818470 35801156 PMC9254717

[17] Dun L Xian-Yi W Xiao-Ying J. Effects of moderate-to-vigorous physical activity on cancer-related fatigue in patients with colorectal cancer: A systematic review and meta-analysis. Arch Med Res. (2020) 51:173–9. doi: 10.1016/j.arcmed.2019.12.015 32111495

[18] Sharma P Hu-Lieskovan S Wargo JA Ribas A. Primary, adaptive, and acquired resistance to cancer immunotherapy. Cell. (2017) 168:707–23. doi: 10.1016/j.cell.2017.01.017 PMC5391692 28187290

[19] Orange ST Jordan AR Odell A Kavanagh O Hicks KM Eaglen T. Acute aerobic exercise-conditioned serum reduces colon cancer cell proliferation *in vitro* through interleukin-6-induced regulation of DNA damage. Int J Cancer. (2022) 151:265–74. doi: 10.1002/ijc.v151.2 PMC9314683 35213038

[20] Rock CL Thomson CA Sullivan KR Howe CL Kushi LH Caan BJ. American Cancer Society nutrition and physical activity guideline for cancer survivors. CA Cancer J Clin. (2022) 72:230–62. doi: 10.3322/caac.21719 35294043

[21] He W Chao J Gu A Wang D. Evaluation of 6-PPD quinone toxicity on lung of male BALB/c mice by quantitative proteomics. Sci Total Environ. (2024) 922:171220. doi: 10.1016/j.scitotenv.2024.171220 38412880

[22] Sun Z Wang J Fan Z Yang Y Meng X Ma Z. Investigating the prognostic role of lncRNAs associated with disulfidptosis-related genes in clear cell renal cell carcinoma. J Gene Med. (2024) 26:e3608. doi: 10.1002/jgm.v26.1 37897262

[23] Zhu C Sun Z Wang J Meng X Ma Z Guo R. Exploring oncogenes for renal clear cell carcinoma based on G protein-coupled receptor-associated genes. Discovery Oncol. (2023) 14:182. doi: 10.1007/s12672-023-00795-z PMC10564696 37816979

[24] Jiang S Yang X Lin Y Liu Y Tran LJ Zhang J. Unveiling Anoikis-related genes: A breakthrough in the prognosis of bladder cancer. J Gene Med. (2024) 26:e3651. doi: 10.1002/jgm.v26.1 38282152

[25] Wang Y Wang X. A pan-cancer analysis of heat-shock protein 90 beta1(HSP90B1) in human tumours. Biomolecules. (2022) 12(10):1377. doi: 10.3390/biom12101377 36291587 PMC9599833

[26] Wang Y Zhu H Xu H Qiu Y Zhu Y Wang X. Senescence-related gene c-Myc affects bladder cancer cell senescence by interacting with HSP90B1 to regulate cisplatin sensitivity. Aging (Albany NY). (2023) 15:7408–23. doi: 10.18632/aging.204863 PMC10457043 37433010

[27] Liang R Hong W Zhang Y Ma D Li J Shi Y. Deep dissection of stemness-related hierarchies in hepatocellular carcinoma. J Transl Med. (2023) 21:631. doi: 10.1186/s12967-023-04425-8 37717019 PMC10505333

[28] Niemira M Collin F Szalkowska A Bielska A Chwialkowska K Reszec J. Molecular signature of subtypes of non-small-cell lung cancer by large-scale transcriptional profiling: identification of key modules and genes by weighted gene co-expression network analysis (WGCNA). Cancers (Basel). (2019) 12(1):37. doi: 10.3390/cancers12010037 31877723 PMC7017323

[29] Li Z Zhou H Xia Z Xia T Du G Franziska SD. HMGA1 augments palbociclib efficacy via PI3K/mTOR signaling in intrahepatic cholangiocarcinoma. biomark Res. (2023) 11:33. doi: 10.1186/s40364-023-00473-w 36978140 PMC10053751

[30] Zhai X Xia Z Du G Zhang X Xia T Ma D. LRP1B suppresses HCC progression through the NCSTN/PI3K/AKT signaling axis and affects doxorubicin resistance. Genes Dis. (2023) 10:2082–96. doi: 10.1016/j.gendis.2022.10.021 PMC10363646 37492741

[31] Zhang H Xia T Xia Z Zhou H Li Z Wang W. KIF18A inactivates hepatic stellate cells and alleviates liver fibrosis through the TTC3/Akt/mTOR pathway. Cell Mol Life Sci. (2024) 81:96. doi: 10.1007/s00018-024-05114-5 38372748 PMC10876760

[32] Zimmerman TA Wang M Lowenthal MS Turko IV Phinney KW. Quantification of transferrin in human serum using both QconCAT and synthetic internal standards. Anal Chem. (2013) 85:10362–8. doi: 10.1021/ac402326v 24074274

[33] Zhai X Zhang H Xia Z Liu M Du G Jiang Z. Oxytocin alleviates liver fibrosis via hepatic macrophages. JHEP Rep. (2024) 6:101032. doi: 10.1016/j.jhepr.2024.101032 38882603 PMC11177191

[34] Qian X Zhang HY Li QL Ma GJ Chen Z Ji XM. Integrated microbiome, metabolome, and proteome analysis identifies a novel interplay among commensal bacteria, metabolites and candidate targets in non-small cell lung cancer. Clin Transl Med. (2022) 12:e947. doi: 10.1002/ctm2.v12.6 35735103 PMC9218934

[35] Wang Y Li C He J Zhao Q Zhou Y Sun H. Multi-omics analysis and experimental validation of the value of monocyte-associated features in prostate cancer prognosis and immunotherapy. Front Immunol. (2024) 15:1426474. doi: 10.3389/fimmu.2024.1426474 38947325 PMC11211272

[36] Zhang J Peng G Chi H Yang J Xie X Song G. CD8 + T-cell marker genes reveal different immune subtypes of oral lichen planus by integrating single-cell RNA-seq and bulk RNA-sequencing. BMC Oral Health. (2023) 23:464. doi: 10.1186/s12903-023-03138-0 37422617 PMC10329325

[37] Wang X Zhao Y Strohmer DF Yang W Xia Z Yu C. The prognostic value of MicroRNAs associated with fatty acid metabolism in head and neck squamous cell carcinoma. Front Genet. (2022) 13:983672. doi: 10.3389/fgene.2022.983672 36110217 PMC9468645

[38] Zhang X Zhuge J Liu J Xia Z Wang H Gao Q. Prognostic signatures of sphingolipids: Understanding the immune landscape and predictive role in immunotherapy response and outcomes of hepatocellular carcinoma. Front Immunol. (2023) 14:1153423. doi: 10.3389/fimmu.2023.1153423 37006285 PMC10063861

[39] Wang Y Zhu H Wang X. Prognosis and immune infiltration analysis of endoplasmic reticulum stress-related genes in bladder urothelial carcinoma. Front Genet. (2022) 13:965100. doi: 10.3389/fgene.2022.965100 36186448 PMC9520708

[40] Ma B Qin L Sun Z Wang J Tran LJ Zhang J. The single-cell evolution trajectory presented different hypoxia heterogeneity to reveal the carcinogenesis of genes in clear cell renal cell carcinoma: Based on multiple omics and real experimental verification. Environ Toxicol. (2024) 39:869–81. doi: 10.1002/tox.24009 37886854

[41] Wang J Zuo Z Yu Z Chen Z Tran LJ Zhang J. Collaborating single-cell and bulk RNA sequencing for comprehensive characterization of the intratumor heterogeneity and prognostic model development for bladder cancer. Aging (Albany NY). (2023) 15:12104–19. doi: 10.18632/aging.205166 PMC10683618 37950728

[42] Wang Y Wang J Liu J Zhu H. Immune-related diagnostic markers for benign prostatic hyperplasia and their potential as drug targets. Front Immunol. (2024) 15:1516362. doi: 10.3389/fimmu.2024.1516362 39703506 PMC11655502

[43] Zhang P Pei S Wu L Xia Z Wang Q Huang X. Integrating multiple machine learning methods to construct glutamine metabolism-related signatures in lung adenocarcinoma. Front Endocrinol (Lausanne). (2023) 14:1196372. doi: 10.3389/fendo.2023.1196372 37265698 PMC10229769

[44] Kakati T Bhattacharyya DK Barah P Kalita JK. Comparison of methods for differential co-expression analysis for disease biomarker prediction. Comput Biol Med. (2019) 113:103380. doi: 10.1016/j.compbiomed.2019.103380 31415946

[45] Deshpande D Chhugani K Chang Y Karlsberg A Loeffler C Zhang J. RNA-seq data science: From raw data to effective interpretation. Front Genet. (2023) 14:997383. doi: 10.3389/fgene.2023.997383 36999049 PMC10043755

[46] D’Souza-Schorey C Chavrier P. ARF proteins: roles in membrane traffic and beyond. Nat Rev Mol Cell Biol. (2006) 7:347–58. doi: 10.1038/nrm1910 16633337

[47] Hashimoto S Furukawa S Hashimoto A Tsutaho A Fukao A Sakamura Y. ARF6 and AMAP1 are major targets of KRAS and TP53 mutations to promote invasion, PD-L1 dynamics, and immune evasion of pancreatic cancer. Proc Natl Acad Sci U.S.A. (2019) 116:17450–9. doi: 10.1073/pnas.1901765116 PMC6717289 31399545

[48] Wee Y Wang J Wilson EC Rich CP Rogers A Tong Z. Tumour-intrinsic endomembrane trafficking by ARF6 shapes an immunosuppressive microenvironment that drives melanomagenesis and response to checkpoint blockade therapy. Nat Commun. (2024) 15:6613. doi: 10.1038/s41467-024-50881-1 39098861 PMC11298541

[49] Luttgenau SM Emming C Wagner T Harms J Guske J Weber K. Pals1 prevents Rac1-dependent colorectal cancer cell metastasis by inhibiting Arf6. Mol Cancer. (2021) 20:74. doi: 10.1186/s12943-021-01354-2 33941200 PMC8094600

[50] Zhang X Hu Y Pan Y Xiong Y Zhang Y Han M. DDR1 promotes hepatocellular carcinoma metastasis through recruiting PSD4 to ARF6. Oncogene. (2022) 41:1821–34. doi: 10.1038/s41388-022-02212-1 PMC8933278 35140331

[51] Song F Zhang Q Lu X Xu T Hu Q Hu X. Rab11-FIP4 interacts with ARF5 to promote cancer stemness in hepatocellular carcinoma. J Physiol Biochem. (2023) 79(4):757–70. doi: 10.1007/s13105-023-00972-2 37458957

[52] Lempicki C Milosavljevic J Laggner C Tealdi S Meyer C Walz G. Discovery of a small molecule with an inhibitory role for RAB11. Int J Mol Sci. (2024) 25(23):13224. doi: 10.3390/ijms252313224 39684933 PMC11642393

[53] O’Sullivan MJ Lindsay AJ. The endosomal recycling pathway-at the crossroads of the cell. Int J Mol Sci. (2020) 21(17):6074. doi: 10.3390/ijms21176074 32842549 PMC7503921

[54] Joseph I Flores J Farrell V Davis J Bianchi-Smak J Feng Q. RAB11A and RAB11B control mitotic spindle function in intestinal epithelial progenitor cells. EMBO Rep. (2023) 24:e56240. doi: 10.15252/embr.202256240 37424454 PMC10481667

[55] Kjos I Vestre K Guadagno NA Borg Distefano M Progida C. Rab and Arf proteins at the crossroad between membrane transport and cytoskeleton dynamics. Biochim Biophys Acta Mol Cell Res. (2018) 1865:1397–409. doi: 10.1016/j.bbamcr.2018.07.009 30021127

[56] Redhai S Boutros M. The role of organelles in intestinal function, physiology, and disease. Trends Cell Biol. (2021) 31:485–99. doi: 10.1016/j.tcb.2021.01.003 33551307

[57] Yin G Lv G Zhang J Jiang H Lai T Yang Y. Early-stage structure-based drug discovery for small GTPases by NMR spectroscopy. Pharmacol Ther. (2022) 236:108110. doi: 10.1016/j.pharmthera.2022.108110 35007659

[58] Gray JL von Delft F Brennan PE. Targeting the small GTPase superfamily through their regulatory proteins. Angew Chem Int Ed Engl. (2020) 59:6342–66. doi: 10.1002/anie.201900585 PMC7204875 30869179

[59] Fiuza-Luces C Valenzuela PL Galvez BG Ramirez M Lopez-Soto A Simpson RJ. The effect of physical exercise on anticancer immunity. Nat Rev Immunol. (2024) 24:282–93. doi: 10.1038/s41577-023-00943-0 37794239

[60] Xiao J Lin H Liu B Xia Z Zhang J Jin J. Decreased S1P and SPHK2 are involved in pancreatic acinar cell injury. biomark Med. (2019) 13:627–37. doi: 10.2217/bmm-2018-0404 31157539

[61] Xiao J Huang K Lin H Xia Z Zhang J Li D. Mogroside II(E) inhibits digestive enzymes via suppression of interleukin 9/interleukin 9 receptor signalling in acute pancreatitis. Front Pharmacol. (2020) 11:859. doi: 10.3389/fphar.2020.00859 32587518 PMC7298197

[62] Furman D Campisi J Verdin E Carrera-Bastos P Targ S Franceschi C. Chronic inflammation in the etiology of disease across the life span. Nat Med. (2019) 25:1822–32. doi: 10.1038/s41591-019-0675-0 PMC7147972 31806905

[63] Llorente A Brokane A Mlynska A Puurand M Sagini K Folkmane S. From sweat to hope: The role of exercise-induced extracellular vesicles in cancer prevention and treatment. J Extracell Vesicles. (2024) 13:e12500. doi: 10.1002/jev2.12500 39183543 PMC11345496

[64] He A Pu Y Jia C Wu M He H Xia Y. The influence of exercise on cancer risk, the tumor microenvironment and the treatment of cancer. Sports Med. (2024) 54:1371–97. doi: 10.1007/s40279-024-02031-2 38687441

